# Soybean Meal Induces Intestinal Inflammation in Zebrafish Larvae

**DOI:** 10.1371/journal.pone.0069983

**Published:** 2013-07-23

**Authors:** Manuel I. Hedrera, Jorge A. Galdames, Maria F. Jimenez-Reyes, Ariel E. Reyes, Ruben Avendaño-Herrera, Jaime Romero, Carmen G. Feijóo

**Affiliations:** 1 Departamento de Ciencias Biologicas, Facultad de Ciencias Biologicas, Universidad Andres Bello, Santiago, Chile; 2 Instituto de Nutrición y Tecnología de los Alimentos, Universidad de Chile, Santiago, Chile; 3 Interdisciplinary Center for Aquiculture Research (INCAR), Concepción, Chile; The University of Plymouth, United Kingdom

## Abstract

The necessary replacement of fish meal with other protein source in diets of commercially important fish has prompted the study of the effect of the inclusion of different vegetable proteins sources on growth performance and on the gastro-intestinal tract. Currently, soybean meal is the primary protein source as a fish meal replacement because of its low price and high availability. Likewise, it is been documented that the ingestion of soybean meal by several fish species, such as salmonids and carp, triggers a type of intestinal inflammation called enteritis. In this paper, we analyzed the effects of the ingestion of soybean meal and two of its components, soy protein and soy saponin, on zebrafish to establish the basis for using zebrafish larvae as a model for fish nutrition. We took advantage of the existence of different transgenic lines, which allowed us to perform *in vivo* analysis. Our results indicated that larvae that were feed with soybean meal developed a clear intestinal inflammation as early as two day after beginning the diet. Moreover, we determined that is not the soy protein present in the diet but the soy saponin that is primarily responsible for triggering the immune response. These findings support the use of zebrafish screening assays to identify novel ingredients that would to improved current fish diets or would formulate new ones.

## Introduction

For more than 50 years, zebrafish has been a model organism primarily used in developmental biology and embryology [1]. Over the last two decades, this fish has become an increasingly popular model organism in other research areas, such as biomedicine [2–4], biotechnology [5–9]. Unlike the fields mentioned above, aquaculture is one of the discipline in which zebrafish is beginning to be considered. In particular, research on immune response, the nutrition and the growth of zebrafish can be expected to produce results applicable to commercially important farmed fish by improving husbandry, survival and formulated feeds, for example.

For the last several decades, aquaculture has grown by approximately 10% annually [10]. This expansion has been accompanied by a progressive increase in the need for fish meal, the base component in the feed formulation for aquatic animals. Soon, the global production of fish meal will not be able to respond to the needs imposed by aquaculture [11–13], it is essential to identify alternatives sources of protein that are economically viable, environmentally friendly, healthy for fishes and capable of at least to sustaining the current aquaculture industry growth rate. Currently, soy products are the foremost candidates to supply fish meal protein; of these, the most commonly used is soybean meal (SBM). Studies in different fish species, such Atlantic salmon, rainbow trout and carp, indicated that the inclusion of SBM in the diet negatively influence the feed ingestion, the intestinal morphology and the immunological function of these fish [14–21]. In these species, SBM triggers an inflammation process in the distal intestine, which is characterized by shorter primary and secondary mucosal folds, an increase in the number of goblet cells [22,23] and the infiltration of macrophages, neutrophils, lymphocytes, eosinophils, immunoglobulin M (IgM) and T cells into the lamina propria, a situation that decrease the capacity of the distal intestine to absorb nutrients [14,24]. These effects proved to be dose–dependent; the worst symptoms were observed at the highest inclusion level (30%), but even the lowest amount of SBM (10%) generates adverse effects [17]. Several studies indicate that it is not the soy protein that is responsible for the off-target effects of SBM. There is evidence that suggest that saponin, an antinutritional factor present in SBM, is principally responsible for enteritis in Atlantic salmon [14,17,24–27]. Although the available evidence indicate that the addition of SBM to fish food has negative effects, the results obtained in the various research conducted are difficult to compare. That variance may be explained by the use of different source of soybean meal, feed formulation or experimental design. However, to fully understand the pathologic processes triggered at a molecular and cellular level by the ingestion of SBM in commercially important fish species is not possible because of a lack of knowledge of the biology of these species. Alternatively, we propose to use a model fish, the zebrafish (*Danio rerio*), instead of analyzing the repercussions of SBM ingestion on a specific fish species. This teleost fish has an especially well known biology, rapid development and easy handling that allows us to perform all analysis *in vivo* and with a high number of specimens per data point [1,28]. In this paper, we propose the zebrafish as a model fish for identifying ingredients that will allow the development of new strategies to improve diet formulation, and thus, the nutrition of farmed fish. Specifically, we demonstrated, for the first time, that soybean meal, as in other fish species, induce an inflammatory process in zebrafish. Moreover, other components present in the soybean meal, such as saponin, are responsible for triggering the inflammation, unlike soy protein that is not. Finally, we demonstrate that the *in vivo* analysis of the inflammatory process by monitoring the infiltration of neutrophils in the intestine, is a simple method that allows the detecting of the effects of different diets early in the inflammation process.

## Results

### Larvae fed with soybean meal developed an intestinal inflammatory process

To evaluate whether the zebrafish develop the same intestinal effects that are triggered in commercially important farm fish by the ingestion of vegetal proteins, primarily soybean, we formulated three different diets. The first diet was a commercial pellet for zebrafish larva (micron, Sera) and was used as a negative control (control); the second diet had fish meal as the principal protein source (100FM) and the third diet contained an inclusion of 50% soybean meal (50SBM) (Table 1). Additionally, we standardized a feeding protocol, where larvae were fed for 4 days with one of the 3 diets analyzed (Figure S1). To confirm whether the larvae actually ate the food administered, they were monitored under a stereoscope on each day of the trial. Because of larval transparency, it was possible to observe the presence of food inside the intestine in more than 85% of individuals daily (Figure S2).

**Table 1:** Formulation of experimental diets.

**gr/Kg**	**100FM**	**50SBM**	**43SP**	**50SS**
**Fish meal (FM)** ^1^	610	250	302.75	606.7
**Soybean meal (SBM)** ^2^	0	500	0	0
**Soy protein isolate (SP)** ^2^	0	0	432.5	0
**Soy Saponin (SS)** ^3^	0	0	0	3.3
**Wheat grain meal**	255	115	129.75	255
**Starch**	45	45	45	45
**Fish oil**	30	60	30	30
**Vitamin mix** ^2^	15	15	15	15
**Mineral mix** ^2^	15	15	15	15
**Cellulose**	30	0	30	30
**Total**	1000	1000	1000	1000

^1^ Exapesca S.A.

^2^ BioMar Chile S.A

^3^ Gushen Biological Technology Group.

**Figure 1:**
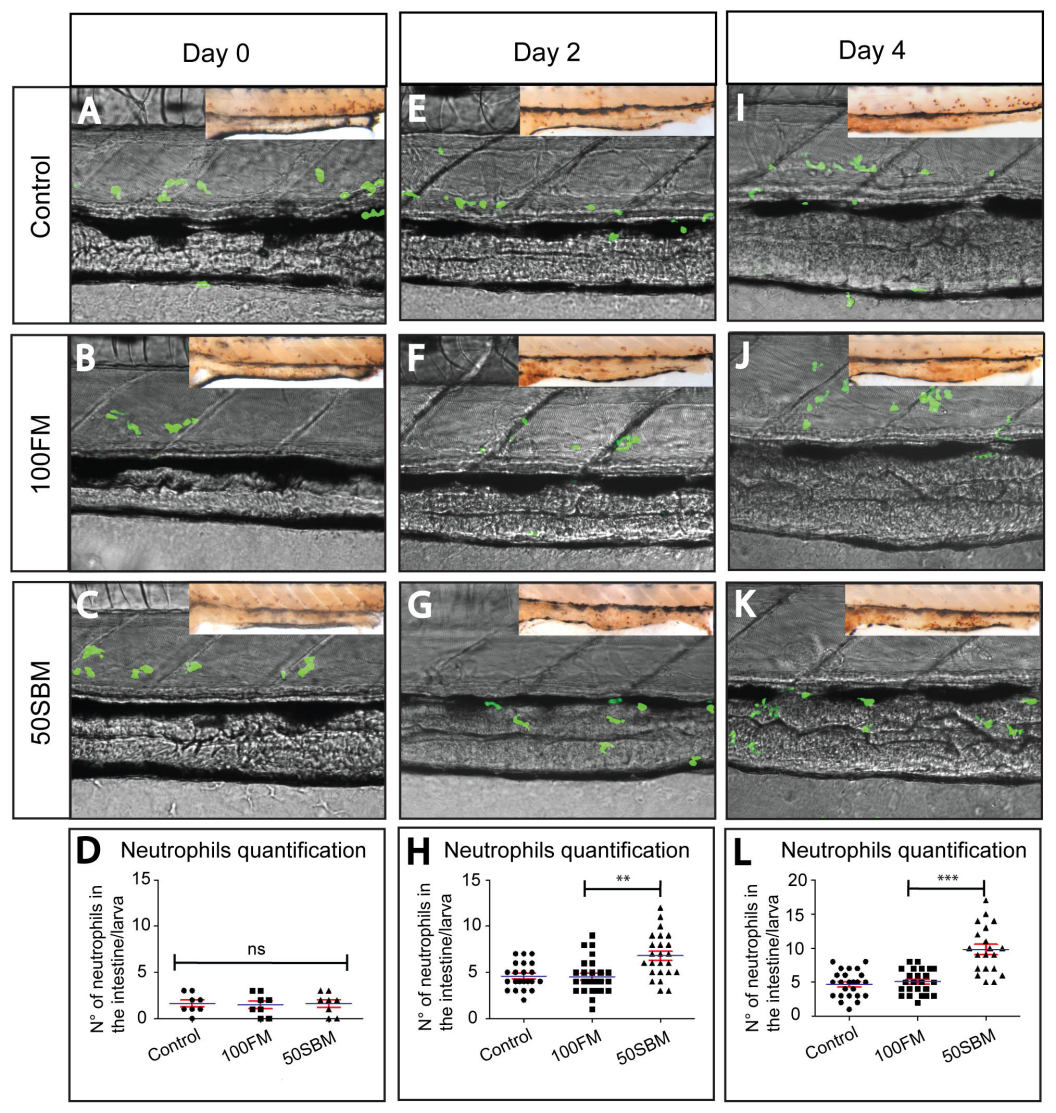
Soybean meal triggers neutrophil migration to the intestine after two days of feeding. Tg(mpx:GFP) transgenic larvae were analyzed *in vivo* by confocal microscopy to monitor neutrophil infiltration into the intestine at day 0 (A–C), 2 (E–G) and 4 (I–K) of feeding with control (pellet) and experimental diets (100FM diet and50SBM diet). (D, H, L) The number of intestine-infiltrated neutrophils was quantified by immunohistochemistry in the same larvae analyzed by confocal microscopy (inset). The results indicated that fish meal did not activate the immune response after 2 or 4 days of feeding. In the case of soybean meal, the number of infiltrated neutrophils was increased from day 2. Five larvae per condition in three different experiments were analyzed per time point by confocal microscopy. For immunohistochemistry analysis, at least 15 larvae per condition per time point in three different experiments were performed. Statistical analysis was conducted using one-way ANOVA. **p value < 0.01; ***pvalue < 0.001.

We monitored the migration of neutrophils to the intestine as a marker of the activation of immune response in this organ. To perform these experiments, we took advantage of the *Tg*(*mpx: GFP*)^*i114*^ transgenic zebrafish line [29], (hereafter referred to as Tg(mpx:GFP)), that expresses GFP under the control of the entire myeloperoxidase regulatory region and that allows the tracking of neutrophils in live animals. Confocal microscopy was used to confirm the presence of this type of granulocyte in the intestine before feeding and at day 2 and 4 of treatment (Figure 1). Both the control diet and 100FM diet did not induce the migration of neutrophils to the intestine at any time point analyzed (Figure 1 A, B, E, F, I J); in contrast, the 50SBM diet triggered the infiltration of immune cells to the intestine since the second day of treatment (Figure 1 C, G, K). To confirm that neutrophils were indeed inside the intestine, we prepared confocal optical sections of larvae after 4 days of treatment, imaging from the left lateral side of the intestine to the right lateral side (Z-axis stack). The highest number of GFP positive cells was detected in the central sections; additionally, some neutrophils were identified in the surrounding peritoneal space and in the skin (Movie S1). This result suggests that, at this stage, the initial inflammation in the intestine has expanded into adjacent tissues.

In some cases, even 12hrs after the last feeding, the food remains in the intestine; considering the autofluorescence that hinders the proper detection of neutrophils, we decided to performed immunohistochemistry to the same larvae analyzed by confocal microscopy (Figure 1 insets) to quantify the number of these immune cells present along the intestine. The mid intestine, excluding the anus, was measured for neutrophils quantification. The results that were obtained did confirm the *in vivo* observations, showing a clear increase in the number of neutrophils present in larvae fed with 50SBM at day 2, that become more robust at day 4. Notably, there was no significant difference between the number of neutrophils present in the intestine of larvae fed with 100FM diet and those larvae fed with control diet (Figure 1 D, H, L).

### Soy saponin and not soy protein is responsible for the intestinal inflammatory process

**Figure 2:**
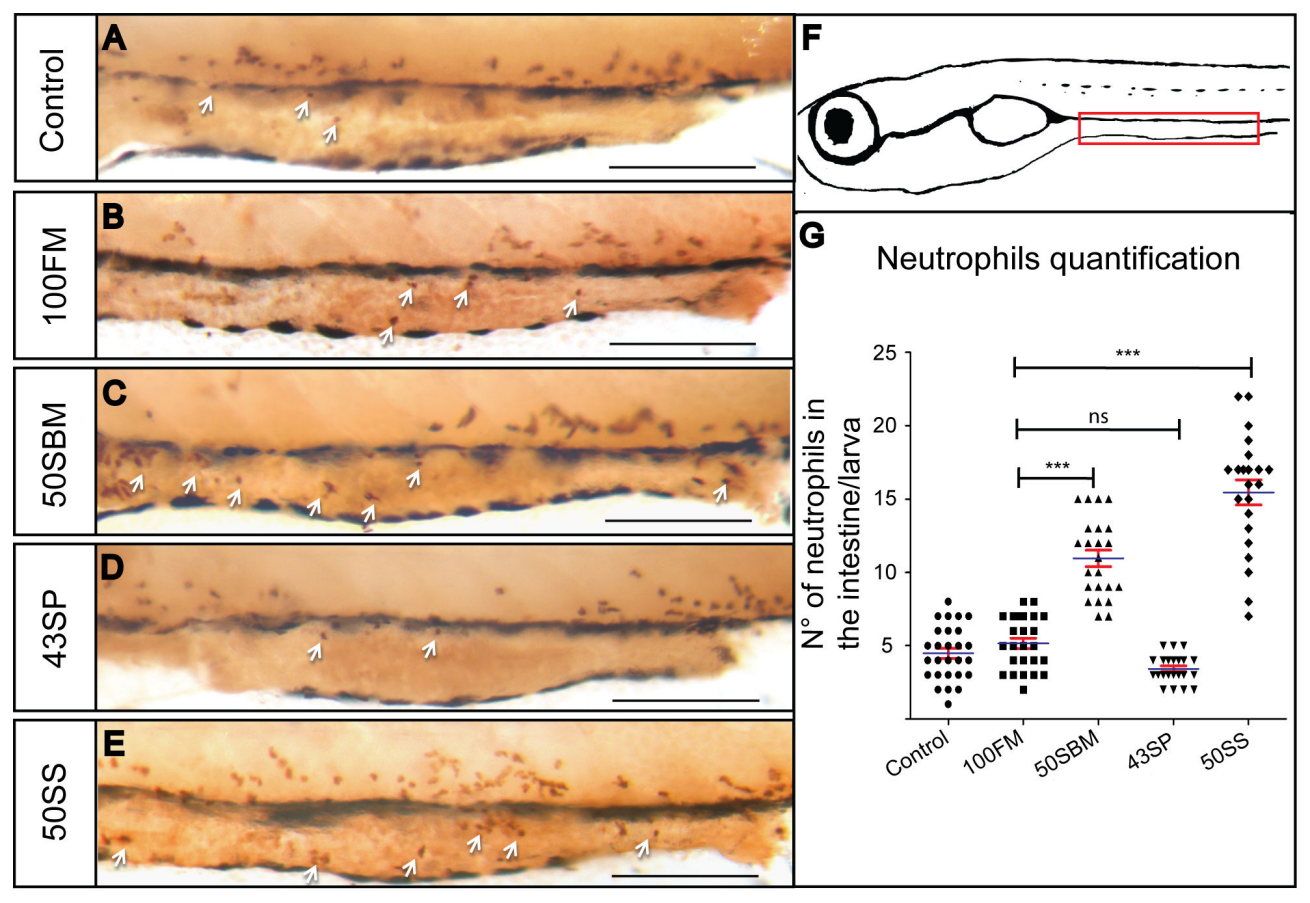
Soy saponin, but not soy protein, increases neutrophil migration to the intestine. Two compounds of soybean meal (50SBM diet) (C), soy protein (43SP diet) (D) and soy saponin (50SS diet) (E), were analyzed to determine their immunological effect. (G) An increase of neutrophils in the intestine (white arrow) was observed after 4 days of feeding in the soy saponin group (and soybean meal), but not in the soy protein group. (F) Scheme representing the intestine territory analyzed (red rectangle). At least 15 larvae per condition were analyzed in three different experiments. Statistical analysis was performed using one-way ANOVA. ns (no significance) *p value < 0.05; ***p value < 0.001. Scale bar = 200µm.

Given that the 50SBM diet triggered an intestinal inflammatory process, we decided to evaluate whether two important components of the soybean meal, soy protein and soy saponin, are responsible for inducing the immune response in the gut. To accomplish this, we fed larvae with 5 diets, the three original; control, 100FM and 50SBM; and two new ones, 43SP and 50SS (Table 1). After feeding, we fixed the larvae to perform immunohistochemistry against GFP in Tg(mpx:GFP), and we quantified the number of neutrophils present in the intestine. Our results indicated that 50SS diet, but not the 43SP, is involved in the inflammatory response triggered by soybean meal (Figure 2). The 50SS diet seems to induce an even more intense inflammation than the 50SBM diet (Figure 2 C, E, G); in turn, the 43SP diet did not show any difference when compared with control or 100FM diets (Figure 2 A, B, D, G). These results support the previous data obtained from the analysis of SBM diet effects in Atlantic salmon [14,17,24–27], thus strengthening our hypothesis that the zebrafish is a good model for analyzing new or alternative aquaculture food components.

**Figure 3:**
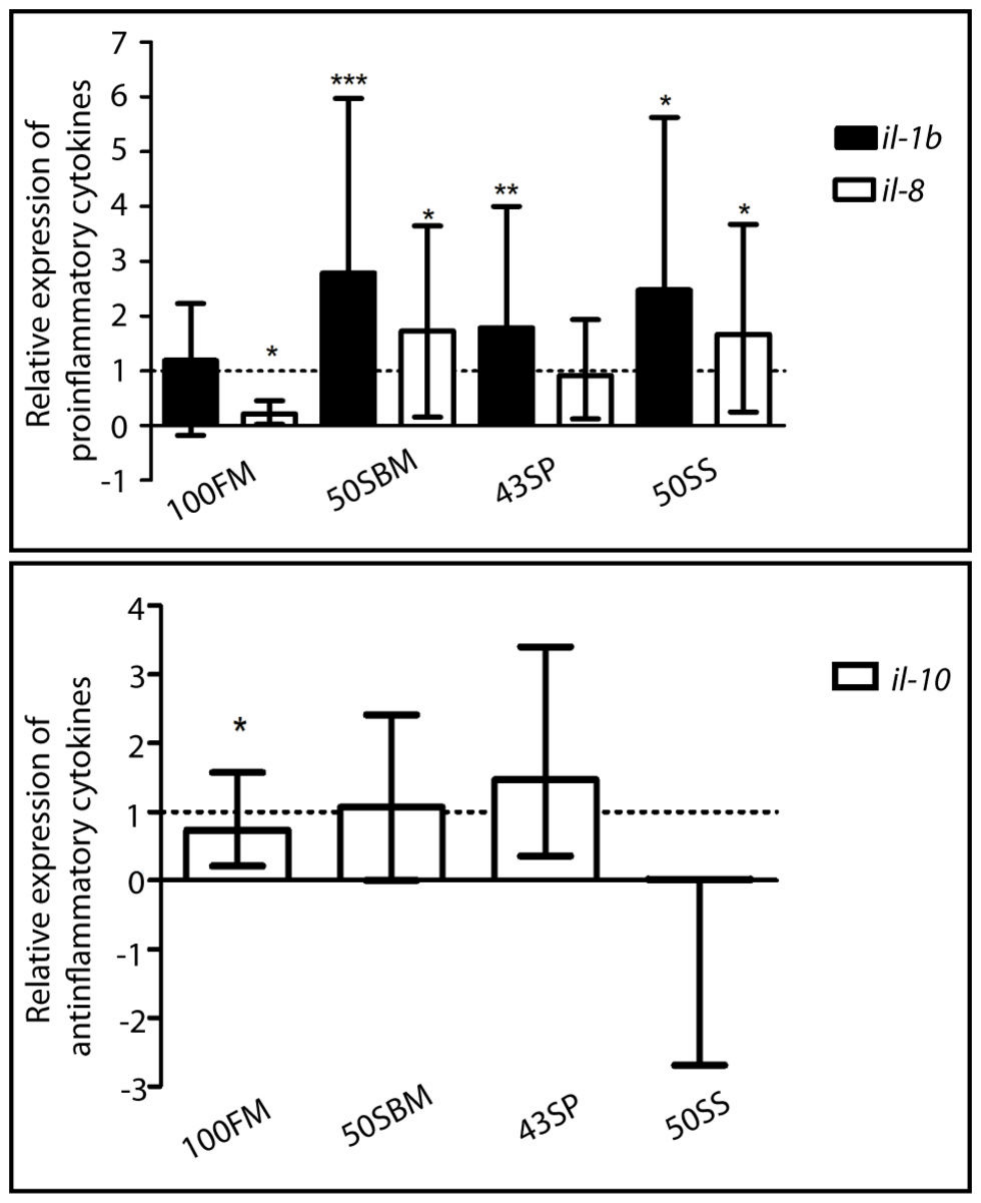
The immunological response triggered by soybean meal and soy saponin is also activated at a molecular level. Transcription levels of pro-inflammatory cytokines, interleukin 1β (*IL-1β*) and interleukin 8 (*il-8*), and the anti-inflammatory cytokine (*il-10*) were quantified by qPCR. Transcription data were normalized to b-actin1 and to the corresponding control. Forty larvae per treatment were analyzed in three different experiments. *p value < 0.05; **p value < 0.01; ***p value < 0.001.

Next and to complement our result we determined by qRT-PCR the transcriptional levels of different cytokines that are key signals during inflammation (Figure 3). The results obtained corroborate our *in vivo* analysis, showing a strong increase in the pro-inflammatory cytokines *IL-1β* and *il-8* mRNA levels in larvae that were fed 50SBM and 50SS diets compared to larvae fed with the control diet. In agreement with the above results, the anti-inflammatory cytokine *il-10* mRNA level remained unchanged.

### After 4 days of feeding the inflammation process has no morphological effects at intestinal tissue

**Figure 4:**
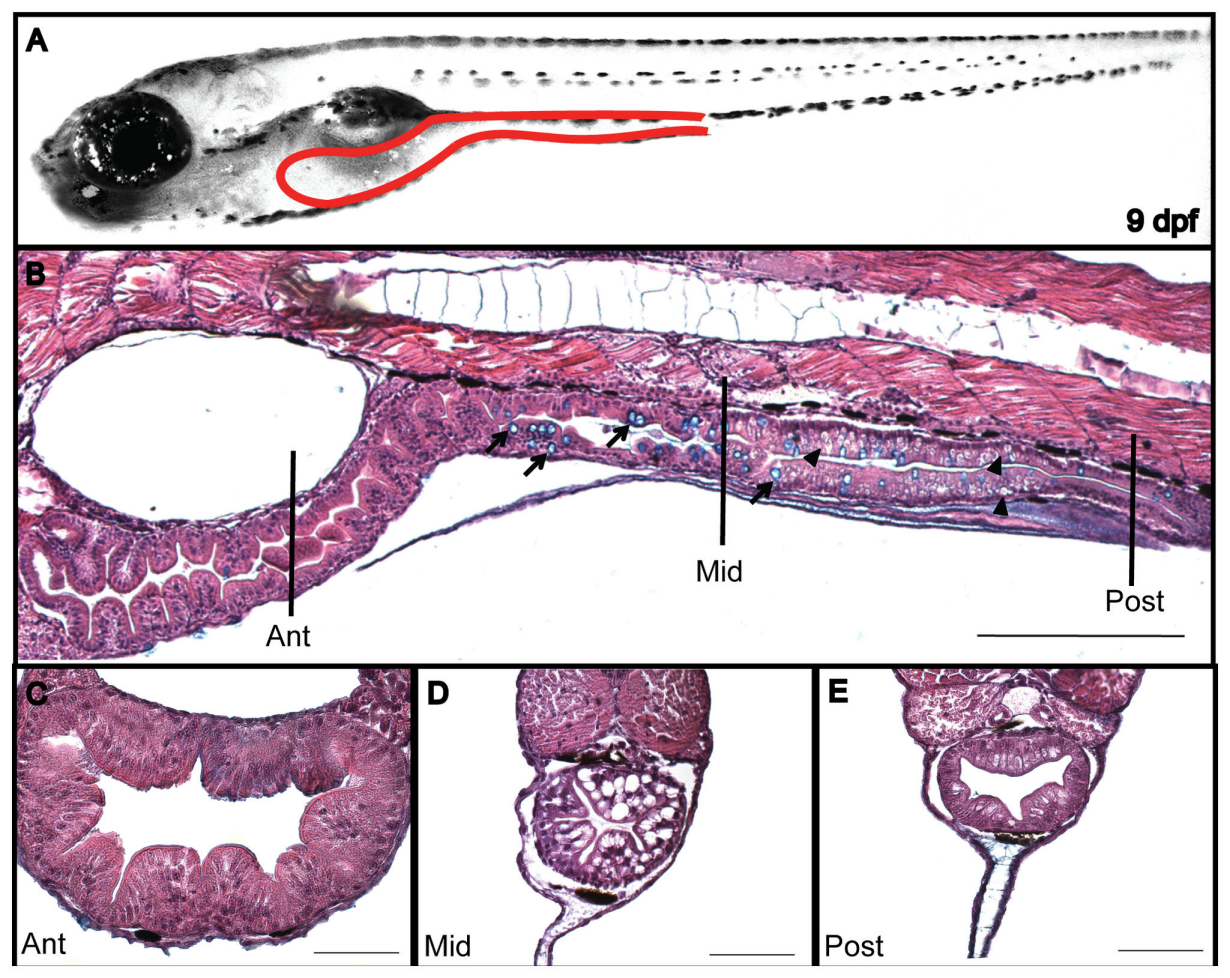
Intestine morphology of a 9dpf larva. (A) Larva lateral view with the intestine delineated (dotted red line). Sagittal (B) and transversal (C–E) paraffin sections stained with H&E and alcian blue. The intestine is divided in 3 different segments: anterior (C) middle (D) and posterior (E). The arrow indicates goblet cells and the arrowhead indicates a supranuclear vesicle. Scale bar = 200µm in B; 50µm in C, D and E.

Performing a histological analysis of the gut is a strategy commonly used to analyze gut effects caused by different components of formulated diets in commercially important fish species [30,31]; therefore, we used different sections (sagittal and transverse) and stains (hematoxylin and eosin (H&E) and alcian blue) to verify the existence of an inflammatory process. First, we characterized the morphology of the intestine of 9dpf larvae fed with either control or 100FM diets for 4 days (Figure 4). In both the sagittal and transverse sections, we observed that the anterior intestine had well-defined folds and a prominent lumen (Figure 4 B, C). In the middle segment, the folds were not as obvious; a high number of goblet cells and supranuclear vesicle were detected, as previously reported [31] (Figure 4 B, D). The posterior region lacked intestinal folds and the lumen was similar to that observed in the middle segment.

**Figure 5:**
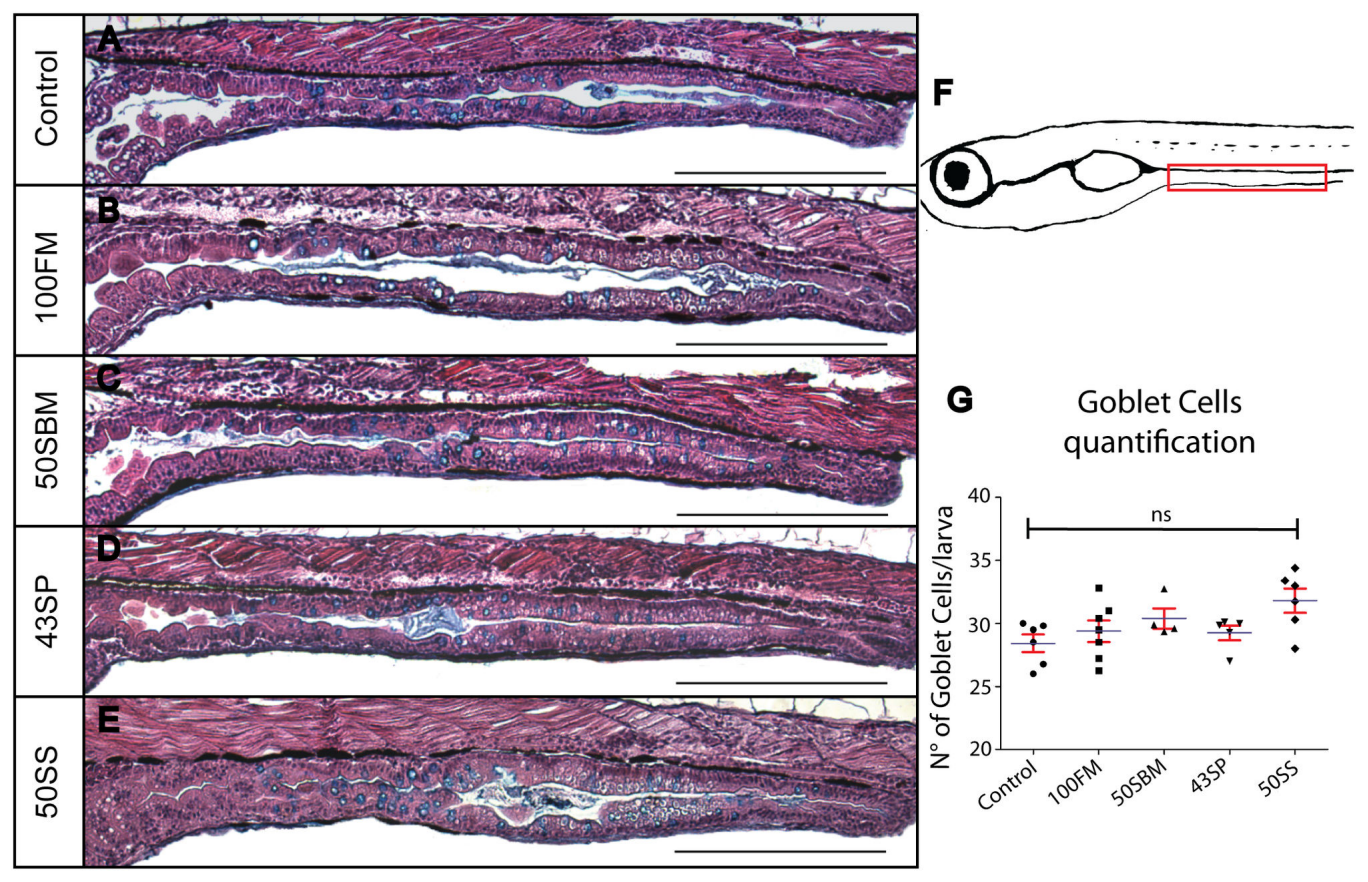
The number of goblet cells in larvae fed with the different diets does notchange after 4 days of treatment. Goblet cells quantification (F) in the intestine was performed in paraffin sections stained with H&E and alcian blue. No change in these mucus-secreting cells was observed between control groups (A) and experimental diets (B–E). At least six larvae were analyzed per condition in three different experiments. Statistical analysis was performed using one-way ANOVA. ns (no significance). Scale bar = 200µm.

The first parameter analyzed was the number of intestinal goblet cells. There are a few articles in fish, zebrafish and Atlantic salmon, that indicate that the number of goblet cells increase in case of intestinal inflammation [30,32,33]. We performed sagittal sectioning of larvae fed with the 5 diets, then stained the sections with H&E to recognize the cell morphology and alcian blue to detect the mucin present in goblet cells. Although it appears that the larvae fed with 50SBM and 50SS diets have more goblet cells (Figure 5 C, E) compared with the larvae fed either with control or 100FM diets (Figure 5 A, B), the statistical analysis of the data indicated that there is no significant difference in the number of goblet cells of larvae intestine among all diets tested.

**Figure 6:**
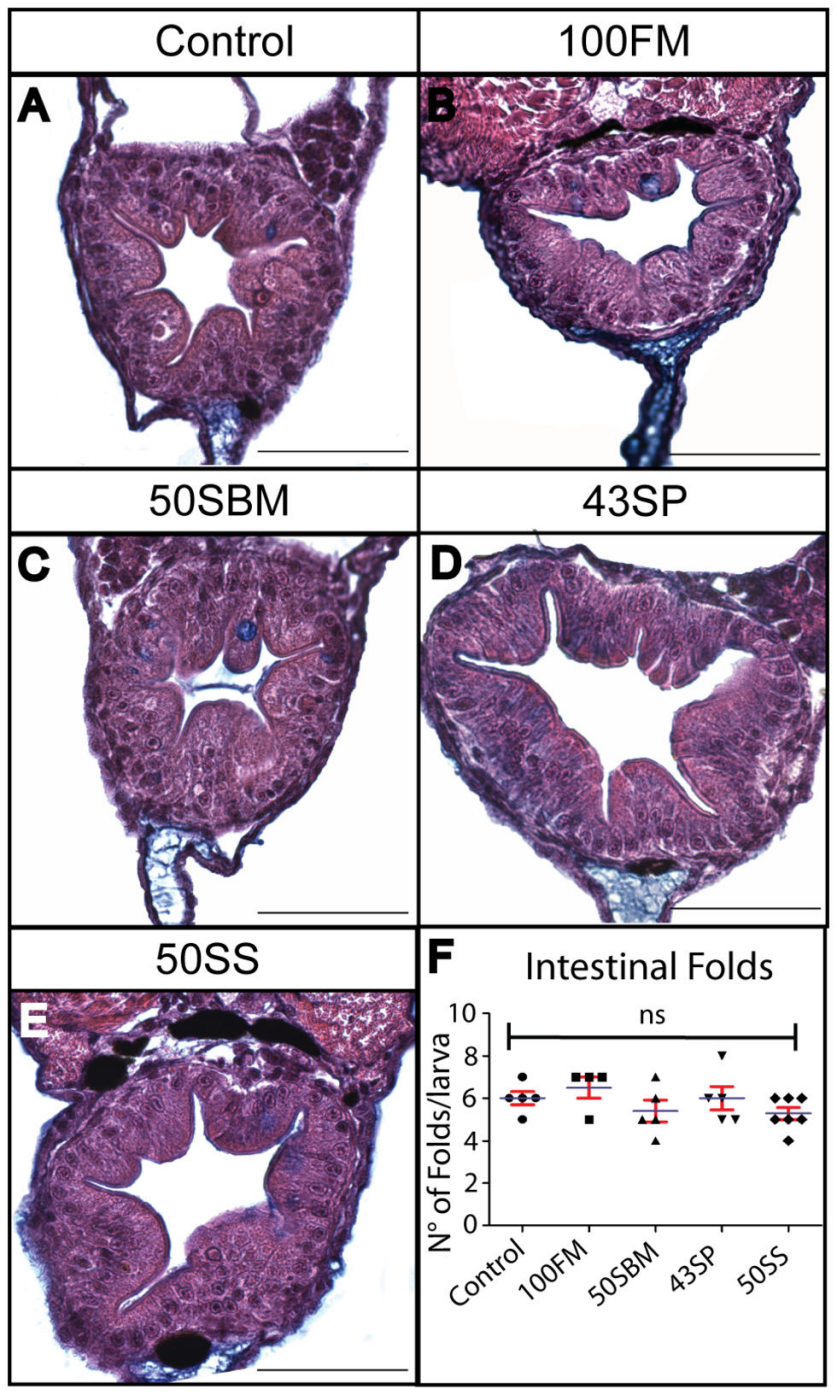
Intestinal folds did not disappear in larvae fed with Soybean meal or soy saponin diets. Transversal section of the mid-intestine, stained with H&E and alcian blue, of larvae fed with the different diets analyzed. The quantification of intestinal folds number in each case indicated that there is no difference between control and experimental diets (F). At least six larvae were analyzed per condition in three different experiments. Statistical analysis was performed using one-way ANOVA. ns (no significance). Scale bar = 50µm.

Another factor used as indicator of inflammation is the presence of intestinal folds. Thus, as inflammation increase, intestinal folds tend to disappear and the gut acquires a smooth appearance [23,31,33]. To determine if the intensity of the immune response generated is sufficient to induce changes in the morphology of intestinal folds, we performed transverse sectioning of the midgut of larvae fed with the five diets. Our analysis indicated that there is no difference in the number, width and length of folds present in the intestine between larvae fed the control diet and larvae fed experimental diets (Figure 6). Additionally, the lumen of the intestine is very similar among the different treatments. These results are in agreement with the results obtained for goblet cells and suggest that 4 days of feeding is an insufficient period to observe the effect of the inflammation triggered by the soybean meal at morphological level.

## Discussion

In this work, we present a new method to analyze the potential intestinal impact that the ingestion of different food ingredients can trigger. We take advantage of the optical transparency of the zebrafish model, which allows robust visual phenotypes to be obtained, making this teleost fish an attractive platform to develop analysis to identify the best, or the less harmful, ingredients to be used in the formulation of diets for aquaculture. As indicated in the literature, and demonstrate in this paper, neutrophils migration towards affected areas correlates with pro-inflammatory cytokine production, thus making transgenic lines ‘‘live indicator’’ of an inflammation process.

The aim of this study was to demonstrate that in zebrafish, as in salmon, trout, carp and other fish species, the ingestion of soybean meal triggers an intestinal inflammation process, thus establishing the basis for using zebrafish larvae as a model for fish nutrition. Although the general inflammatory effects observed in this teleost fish are the same effects reported for other fish species, it seems that there are some differences in the tolerance of zebrafish soybean meal ingestion over other fish, such as Atlantic salmon. It has been reported that even the inclusion of 10% SBM in the diet for Atlantic salmon produces detrimental inflammatory effects at the intestinal level [30]. This observation contrasts with that observed in zebrafish, where with an inclusion of 50% SBM, the inflammatory effect are not detected by histological methods, a technique widely used for detecting inflammatory effects in the intestine in salmonids. However, and perhaps most similar to zebrafish, is that rainbow trout have a higher tolerance to SBM; the inclusion of more than 50% did not trigger an overly negative response [34,35]. This difference with other fish would not present a problem for the use of zebrafish in testing ingredients for diets; rather this difference seems to be an advantage. This advantage is because the ingredients that have adverse effects in the intestine in zebrafish have a high probability of also having negative effects on most “sensitive” fish, such as Atlantic salmon, at least as far as vegetable protein is concerned.

In commercially important fish species, almost all studies that evaluate the impact of diet ingredients on the rate of intestinal inflammation that they trigger take a long time, usually approximately 10 days of feeding in the case of soybean meal diets. This long evaluation time is due to the strategy utilized to analyze the effects, which are primarily morphological changes at the gut, such as length of mucosal fold, the number of goblet cells and the size of subepitelial mucosa [31]. Our results demonstrate that the inflammation process begins long before the effects are observed at histological level. Identical results were also noted by Oehlers et al. [23] in a model of chemically induced inflammatory bowel disease (IBD). After 5 days of treatment, they observed neither an increase in goblet cells nor changes in gut morphology, although the evidence presented demonstrates the existence of intestinal inflammation. Similarly, the work developed in Atlantic salmon indicates that the immune response is activated early following the ingestion of soybean meal [36–38]. These results give greater relevance to our *in vivo* analysis because they allow the early detection of the inflammatory process. The proposed method not only allows for the early detection of the inflammatory process but also facilitates the analysis of many ingredients in a very short time, constituting an important competitive advantage for the aquaculture industry. Likewise, this method is not restricted to different types and qualities of soybean meal but also may be used for the analysis of a wide range of ingredients, from new sources of vegetable protein, to immunostimulant, and to compounds that are able to reverse the inflammatory effects triggered by vegetable protein.

Ours results confirm the hypothesis that the zebrafish recapitulates the same inflammatory effects of the ingestion of different food ingredients as other fish species, at least at the intestine. Thus, this fish is positioned as an advantageous model for carrying out relatively large-scale screens to evaluate intestinal effects of new ingredients that can be incorporated into the formulation of food for aquaculture.

## Materials and Methods

### Ethics Statement

All animals subjected to experimentation were anesthetized, and procedures complied with the guidelines of the Animal Ethics Committees of the Universidad Andres, Bello, which approved this study.

### Zebrafish strains and maintenance

Zebrafish were maintained and raised in our facility according to standard protocols [39]. The following strains of fish were used in this study: Tab 5 (wild type), *Tg*(*mpx: GFP*)^i114^ [29]. All embryos were collected by natural spawning, staged according to Kimmel et al. [28] and raised at 28°C in E3 medium (5 mM NaCl, 0.17 mM KCl, 0.33 mM CaCl_2_, 0.33 mM MgSO_4_, without methylene blue, equilibrated to pH 7.0) in Petri dishes, as described previously [40]. Embryonic and larval ages are expressed in hours post fertilization (hpf) or days post fertilization (dpf).

### Diets

Four experimental diets were formulated: 100FM, 50SBM, 43SP and 50SS (Table 1). As a negative control, a commercial fish pellet was used (Sera micron); this control diet did not contain any soybean meal (SBM). In the case of 100FM diet, the major ingredients were the following: fish meal (protein content above 70%), fish oil and wheat grain mail (protein content above 17%). The 50SBM and 43SP diets were formulated to contain 50% and 43% defatted SBM and SP isolate (protein content 90%) inclusion level, respectively. In the case of the 50SS diet, the amount of soy saponin concentrate (90% purity) added was similar to the level of saponin expected to be found in the 50SBM diet, that is 3.3g/kg (considering that defatted SBM has 0.6%, 6g/kg, of saponin). Diets were supplemented with a standard vitamin and mineral premix. Feed was produced as extruded 2mm sinking pellets and then crushed in a mortar and sieved with a mesh of 75 microns to obtain a particle size that is suitable for consumption by zebrafish larvae.

### Feeding strategy

Forty-five larvae of 5dpf (15 larvae in triplicate) were maintained in 80ml fish water (water from the aquarium system) in a 100ml beaker in the fish facility at 28^°^C. Larvae were fed twice a day, with an interval of at least 6hrs between feedings, for 4 days until 8dpf. During each feeding, larvae were in contact with the food for 1hr and then the medium was replaced for new one. The last feed was conducted at least 14hrs before fixing to promote intestine emptying (Figure S1). After fixing (at 9dpf), larvae were processed according to the different analysis (qPCR, histology or immunohistochemistry).

### Confocal analysis

Five live larvae per condition were anesthetized in tricaine 4% (Sigma) after 2 and 4 days of feeding to determine the number of neutrophils present in the intestine. The experiment was performed at least three different times.

### Histological section

For histology, 9dpf larvae were fixed in Bouin’s solution during 3hrs at RT and were mounted in SeaPlaque 3% agarose (LAFKEN cat: FER/00A200). The blocks of larvae were dehydrated through a standard ethanol series to 100%, were embedded in paraffin [41] and sectioned at 5µm intervals for staining with hematoxylin & eosin (H&E) (Merck, cat: 1.05174.1000; Scharlau, cat: EO0025), and/or alcian blue (Merck cat: 1.05234.0010).

The quantification of goblet cells was performed as follow: Four sagital section from 5µm each were obtained per larva. The goblet cell count was performed in a delimited region in the mid gut (red rectangle in Figure 5 G). The average of the number of goblet cells in the four sections correspond to the cell number in the intestine of one individual; at least six larvae were analyzed per condition in three different experiments. In the case of the number of intestinal folds, ten transversal section of 5µm each (a total of 50µm) were analyzed per larva. The average of the number intestinal folds in the ten sections correspond to the folds number in the intestine of one larva, at least six individuals were analyzed per condition in three different experiments.

### Immunohistochemistry and neutrophils quantification

Immunohistochemetry was essentially preformed as previously described [42]. Briefly, larvae were fixed for 1hr in 4% paraformaldehyde/phosphate buffered saline (PBS), then rinsed with PBS-Tween, dehydrated in 100% methanol and stored at -20°C until processed. The following antibodies were used: rabbit anti-GFP (Invitrogen cat: A11122) and anti-rabbit peroxidase (Sigma, cat: A8275).

The quantification of neutrophils present in the intestine was performed in whole mount larvae, for this purpose a region in the midgut was defined (red rectangle in Figure 2F). At least 15 larvae were analyzed per condition in three different experiments.

### qPCR

Larvae fed with control and experimental diets were sampled at the end of the treatment (9dpf) for total RNA extraction. Samples included 40 larvae per treatment and the experiment was done at least three times. Total RNA extraction was obtained using Trizol Reagent (Invitrogen, cat: 15596-026), according to the manufacturer’s instructions. cDNAs were synthesized from RNA samples in a reverse transcription reaction using Super Script II RT (Invitrogen, cat: 100004925), according to the manufacturer’s instructions and using oligo-dt primers.

Real time PCR was performed following the method described by Rawls et al. [43]. Each gene was tested in octuplicate and verified for non-specificity using melting curves (primers sequence in table 2). The mean Ct values from each sample were normalized against the mean Ct value of a reference gene (β-actin1, housekeeping gene). The relative quantification of each gene was obtained with the Pfaffl method and the REST 2009 software (Qiagen). This software includes a statistical test to determine accuracy of relative expression, which is complex because ratio distributions do not have a standard deviation. REST 2009 software overcomes this limitation by using simple statistical randomization test that uses the permuted expression data rather than the raw Ct values entered by the user [44].

**Table 2:** Primer sequences used for amplification of specific gene production with the RT-qPCR technique.

**Gene**	**Forward Primer**	**Reverse Primer**	**Amplicon (pb)**
***il-1β***	TGGACTTCGCAGCACAAAATG	GTTCACTTCACGCTCTTGGATG	150
***il-8***	TGTGTTATTGTTTTCCTGGCATTTC	GCGACAGCGTGGATCTACAG	81
***il-10***	CACTGAACGAAAGTTTGCCTTAAC	TGGAAATGCATCTGGCTTTG	120
***β-actin1***	GCCAACAGAGAGAAGATGACACAG	CAGGAAGGAAGGCTGGAAGAG	110

### Statistics and imaging

The study was performed as a random single factor experiment investigating the impact of four diets on the intestinal health of zebrafish larvae. We used a non-parametric test, the Kruskall-Wallis one way ANOVA test, to analyze our data. The data were normally distributed (analyzed by the D´Agostino and Pearson normality test) and the variance was not homogenous (analyzed by the Barlett’s test). All the analysis were performed using Prism 4 (GraphPad Software). The significance level was set to P<0.05.

Photographs were taken in an Olympus SZX16 stereoscope with a QImaging MicroPublisher 5.0 RVT camera or an Olympus IX 81 microscope with a MicroPublisher 3,3 RTV camera. Confocal images were acquired with Olympus FluoView FV1000 Spectral Confocal Microscope (software version 2.1). Images were processed with Photoshop CS4 or ImageJ 1.44o. For all the experiments described, the images shown are representative of the effects observed in at least 70% of the individuals.

## Supporting Information

Figure S1 Feeding strategy scheme. (TIFF)

Figure S2 Monitoring larvae food ingestion. Lateral view of a 7dpf larva before feeding (A) and after food ingestion (B, C). Food ingestion can be easily verified by observing food presence in the gut. (D) Quantification of the number of larvae that eat in every feeding.
(TIFF)

Movie S1 Stack on the Z-axis of the midgut of fish fed with soybean meal. Lateral view of a mid-intestine section, from right to left, after 4 days of treatment. As we move towards the center of the bowel, it can be observed that the largest number of neutrophils is located in this zone. The amount of these granulocytes decreases when we move into regions that are more superficial.
(AVI)
