# Supplementary figures and images for: Soybean Meal Induces Intestinal Inflammation in Zebrafish Larvae
Source: PLoS One. 2013 Jul 23;8(7):e69983. doi: 10.1371/journal.pone.0069983 (PMC3720926; doi:10.1371/journal.pone.0069983)

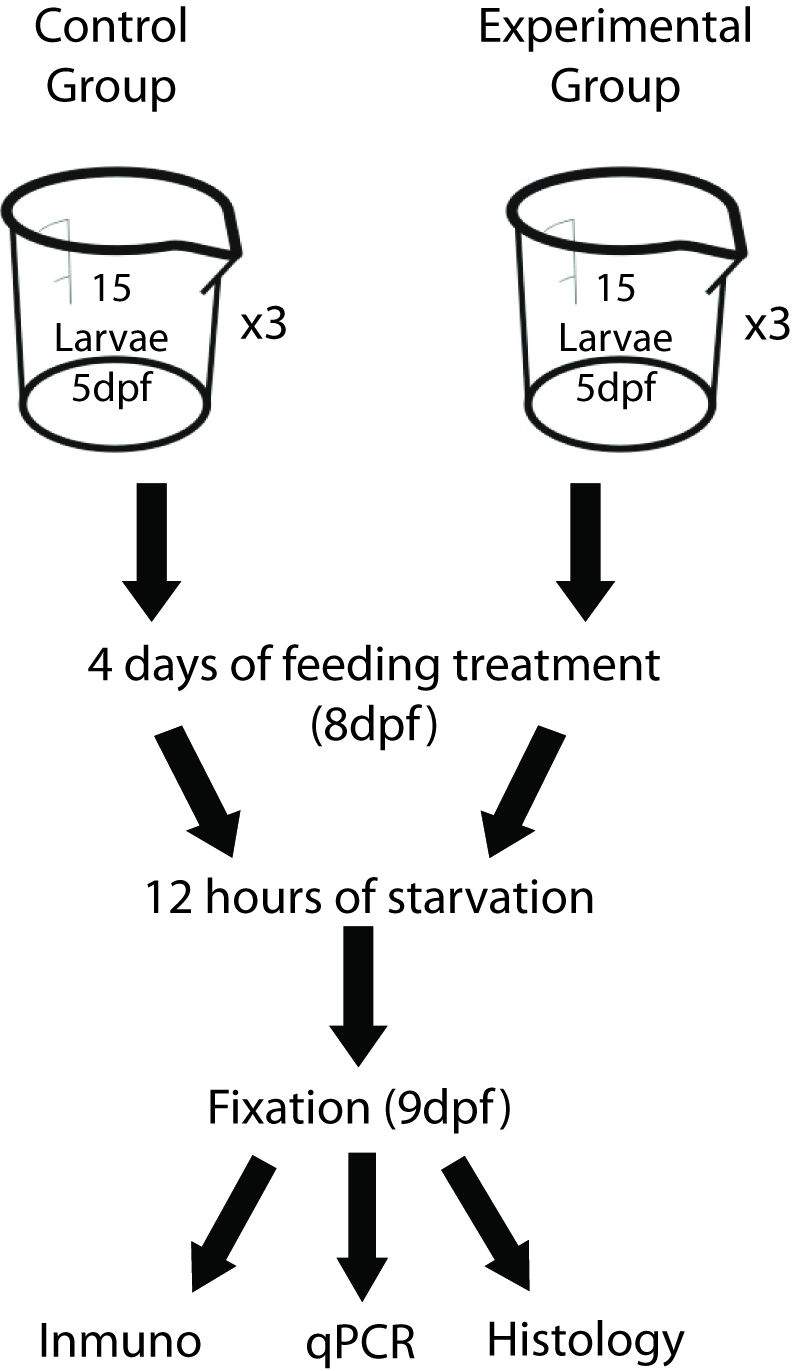

Supplement: Figure S1 — (TIFF) [file pone.0069983.s001.tiff]

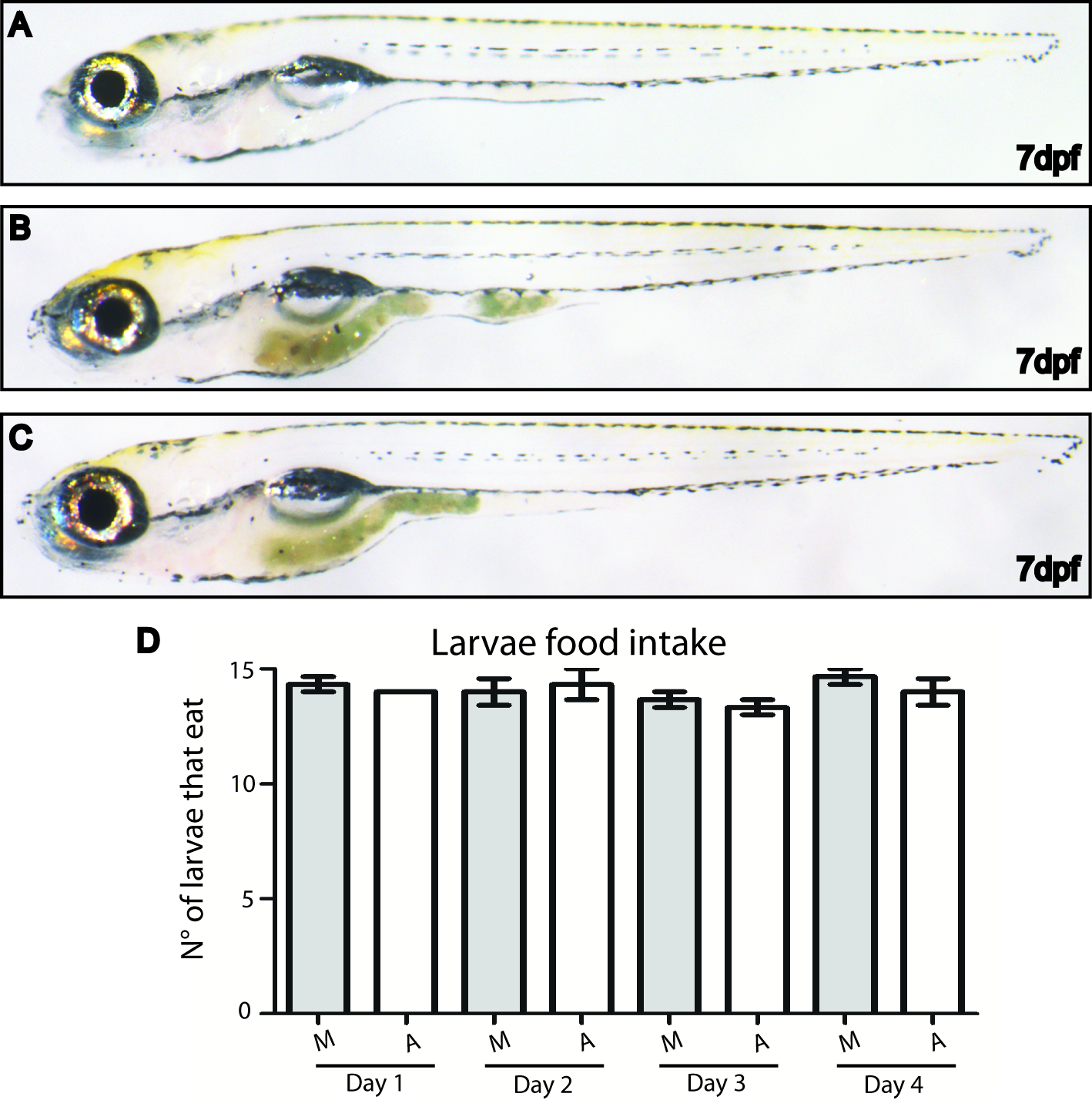

Supplement: Figure S2 — Lateral view of a 7dpf larva before feeding (A) and after food ingestion (B, C). Food ingestion can be easily verified by observing food presence in the gut. (D) Quantification of the number of larvae that eat in every feeding. (TIFF) [file pone.0069983.s002.tiff]
